# Electrochemical Detection of Olivetol Based on Poly(L-Serine) Film Layered Copper Oxide Modified Carbon Paste Electrode (p-L-Serine/CuO/CPE)

**DOI:** 10.3390/nano13010070

**Published:** 2022-12-23

**Authors:** Zongyi You, Yi Zhang, Shengwen Duan, Liangliang Liu

**Affiliations:** Institute of Bast Fiber Crops, Chinese Academy of Agricultural Sciences, Changsha 410205, China

**Keywords:** carbon paste electrode, copper oxide nanoparticles, electrochemical sensor, L-serine, olivetol

## Abstract

Olivetol is an important polyphenol compound and intermediate in the synthesis of cannabinoids possessing many types of biological activities. A facile electrochemical sensor for olivetol was fabricated based on p-L-serine, and copper oxide (CuO) nanoparticles modified carbon paste electrode (p-L-serine/CuO/CPE). The proposed p-L-serine/CuO/CPE was applied to the electrochemical detection of olivetol by cyclic voltammetry (CV) and differential pulse voltammetric (DPV). Through the characterizations of materials and modified electrodes, the p-L-serine/CuO/CPE exhibited enhanced electrochemical signals for olivetol compared to bare CPE and CuO/CPE in both CV and DPV methods. Under the optimized conditions, the proposed p-L-serine/CuO/CPE showed a good quantitative analysis ability and a wide analysis range from 20 to 100 μmol L^−1^ of olivetol with a limit of detection of 1.04 μmol L^−1^. Based on the reproducibility, repeatability, and stability exhibited by this fabricated sensor and the cheap and accessible raw materials, the p-L-serine/CuO/CPE became a novel determination choice for olivetol in the electrochemical method with the advantages of being cost-effective and convenient.

## 1. Introduction

Olivetol, known as 3, 5-dihydroxypentylene, was a natural polyphenol compound that could be found in lichens, some insects, and many plants [1]. It was also an important pharmaceutical intermediate in the synthesis of cannabinoids [2,3]. Olivetol possesses many types of biological activities, including antioxidant, anti-inflammatory, anti-tumor, anti-biotic, anti-cholinergic, antibacterial, and anti-hypertension effects on the human body [4]. It also has therapeutic effects on cardiovascular, diabetes, and other diseases [1,5].

Typical detection methods of olivetol were mainly high-performance liquid chromatography (HPLC), gas chromatography (GC), and colorimetry [6,7]. However, these kinds of techniques had limitations in the experiment cost, time, labor, and speed [8]. Thus, the exploration of a simple, rapid, and accurate technique could expand the detection ability for olivetol in various scenarios.

In recent decades, electrochemical analysis and sensors gained much attention owing to the advantages like portability, low cost, simplicity, rapid response, etc. [9,10]. In order to fabricate various electrochemical sensors, many materials could be applied as the working electrodes [11]. Among them, carbon paste was widely used due to its low cost, easy assembly, strong adjustability, and facile modification of the electrode surface [12]. However, the sensitivity and selectivity of bare carbon paste electrodes (CPE) were limited. So as to prevail over these characteristics, the surface of the bare carbon paste electrode needed to be modified using active materials by immobilization, polymerization, doping, or other methods [13].

For this purpose, electrochemical polymerization was one of the most convenient techniques resulting in a thin polymer film in the redox reaction, which was more uniform and adherent on the surface [14,15]. Many monomers were used for electrochemical polymerization due to the high electron transfer efficiency and low cost, such as dyes, amino acids, and so on [16,17]. L-serine was an amino acid containing polar hydroxyl groups. It played a significant role in maintaining human health and was rich in eggs, fish, and soy [18]. L-serine could be polymerized to form a thin layer on the surface of the electrode [19]. The electropolymerized layer of L-serine (p-L-serine) was frequently applied for electrode modification because it often exhibited enhanced catalytic activity in the sensing of many analytes [19,20,21,22,23]. So far, several reports have investigated the application of p-L-serine on modified electrochemical sensors [22,24,25].

To the best of our knowledge, there was a rare report of the electrochemical sensor based on CPE for the determination of olivetol. In the present study, copper oxide (CuO) nanoparticles were mixed in the preparation of a modified carbon paste electrode (CuO/CPE), and p-L-serine modified CuO/CPE (p-L-serine/CuO/CPE) was developed through electrochemical polymerization. The p-L-serine/CuO/CPE was applied for the determination of olivetol in both cyclic voltammetric (CV) and differential pulse voltammetric (DPV) methods. The modified electrode exhibited improved performance compared to bare CPE in sensitivity and stability.

## 2. Materials and Methods

### 2.1. Reagents

CuSO_4_·5H_2_O, L-serine, olivetol, sodium hydroxide, paraffin oil, and graphite were obtained from Sinopharm Chemical Reagent Co., Ltd. (Shanghai, China). The double distilled water was supplied for the preparation of aqueous solutions in experiments. All reagents were of analytical grade and used as received.

### 2.2. Synthesis of Copper Oxide (CuO) Nanoparticles

As Afzali reported, the CuO nanoparticles were synthesized with some modifications [26]. 4.0 g of CuSO_4_·5H_2_O was dissolved in 100 mL of water, forming a transparent blue solution. Then, 100 mL of NaOH solution (0.3 mol/L) was slowly added into the copper sulfate solution under magnetic stirring at 80 °C. The pH value of this mixture would be adjusted to around 12, and dark brown precipitates would appear. After the incubation at 80 °C for 2 h, the mixture was washed with water by centrifugation. Finally, the product was dried under 105 °C for 8 h in the oven and ground into powders in a mortar for further use. The CuO nanoparticles were characterized by scanning electron microscope (SEM, Apreo 2 Thermo Scientific, Waltham, MA, USA) and X-ray Diffraction (XRD, Rigaku SmartLab 9, Tokyo, Japan) using Cu-Kα radiation at 40 kV and 40 mA.

### 2.3. Preparation of Carbon Paste Electrode (CPE)

The CPE was prepared according to Ghalkhani’s report with some modifications [27]. 70.0% (*w*/*w*) of graphite powder and 30.0% (*w*/*w*) of paraffin oil were hand mixed in an agate mortar until a homogenous paste was obtained. Some amount of this paste was transferred and firmly packed into a cavity at the end of a Teflon tube (3.0 mm inner diameter and 2.0 mm long). A copper wire was fixed in the tube, which connected to the paste, to provide an external electrical contact. Before the usage of CPE, a small portion of the paste was squeezed out, and the surface was polished with weighing paper.

### 2.4. Preparation of Modified CPE

Copper oxide modified carbon paste electrode (CuO/CPE) was prepared as follow. 65.0% (*w*/*w*) of graphite powder, 10.0% (*w*/*w*) of CuO nanoparticles, and 25.0% (*w*/*w*) of paraffin oil were hands mixed adequately in an agate mortar and pestle for 30 min until a thick homogenous paste was obtained. The paste was packed into a cavity at the end of a Teflon tube and treated as the same procedure.

The p-L-serine/CuO/CPE was fabricated by an electrochemical polymerization process using cyclic voltammetry. The CuO/CPE was immersed in 0.2 mol L^−1^ phosphate buffer solution (pH 6.5) containing 1.0 mmol L^−1^ of L-serine and scanned between −0.6 and 1.8 V for 20 cycles. Finally, the electrode surface was washed with water to remove the physically adsorbed L-serine.

### 2.5. Electrochemical Measurement

The electrochemical measurements were carried out by using a CHI 660E electrochemical workstation (Shanghai Chenhua Co., Ltd., Shanghai, China) in the three electrodes system. The CPE and modified CPE were used as the working electrode (3.0 mm diameter). A platinum wire was applied as the auxiliary electrode, and an Ag/AgCl was used as the reference electrode.

The cyclic voltammetry (CV), differential pulse voltammetry (DPV), and electrochemical impedance spectroscopy (EIS) scan modes were applied, and the corresponding voltammograms were recorded. The electrochemical determination of olivetol was performed in an electrolytic cell containing a supporting electrolyte solution (0.01 mol L^−1^ of phosphate buffer saline containing 0.005 mol L^−1^ of potassium chloride, pH 8.0). CV and DPV were recorded from 0 V to 1.0 V with a scan rate of 0.05 V s^−1^. The amplitude was set at 0.05 V for DPV. EIS was scanned in 5.0 mmol L^−1^ of K_3_[Fe(CN)_6_]/K_4_[Fe(CN)_6_] and 0.1 mol L^−1^ of potassium chloride. The potential was set at 0.283 V. The amplitude was set at 0.005 V with a frequency range of 0.01 to 10^5^ Hz. All experiments were carried out in three duplicates at 25 ± 2 °C. After each measurement, the modified electrode was transferred to the water by stirring for 20 s to obtain a renewed electrode surface.

## 3. Results and Discussion

### 3.1. Characterization of CuO

**Figure 1:**
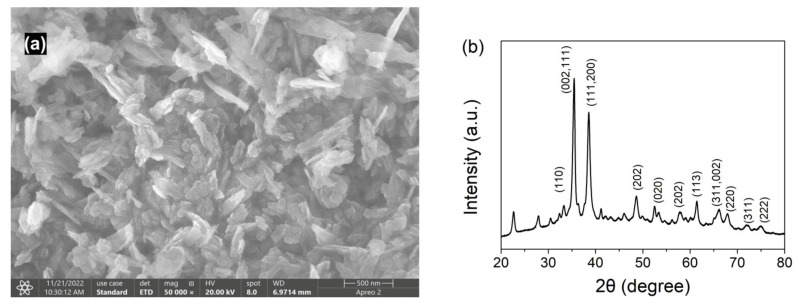
(**a**) SEM image of CuO nanoparticles; (**b**) XRD pattern of CuO nanoparticles.

The SEM image and XRD pattern of CuO nanoparticles are presented in Figure 1. The SEM photograph (Figure 1a) showed the morphology of CuO nanoparticles was agglomerated and irregular in shape, and the size ranged from 50 nm to 100 nm. The diffraction peaks obtained in the XRD pattern of CuO nanoparticles (Figure 1b) at the 2θ values of 33.2°, 35.4°, 38.6°, 48.6°, 52.4°, 57.9°, 61.5°, 66.3°, 67.9°, 72.2°, and 75.2° could be attributed to (110), (111/002), (111/200), (202), (020), (202), (113), (311/002), (220), (311), and (222) planes indicating the monoclinic lattice structure of CuO nanoparticles through the comparing with standard card (JCPDS file: 05-0661) [28,29].

### 3.2. Electropolymerization of L-Serine on the CuO/CPE

**Figure 2:**
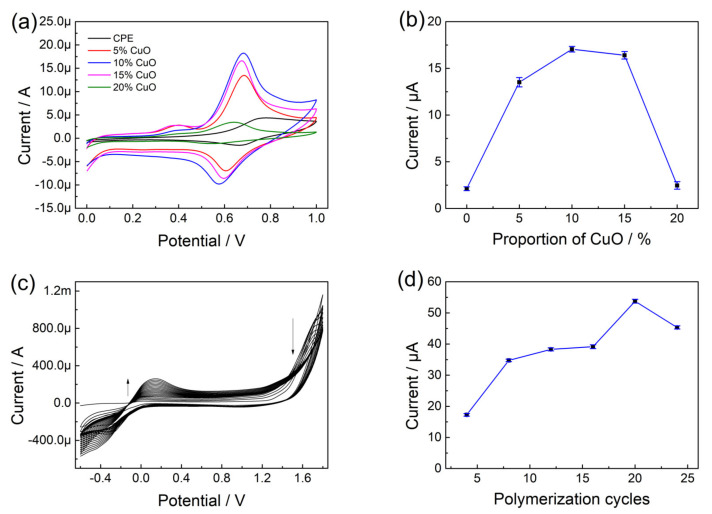
(**a**) CV of CuO/CPE prepared by different proportions of CuO nanoparticles in olivetol solution; (**b**) Effect of proportion of CuO nanoparticles on peak currents of electrodes in olivetol; (**c**) CV of preparation of p-L-serine/CuO/CPE; (**d**) Effect of polymerization cycles on peak currents of electrodes in olivetol.

To optimize the proportion of CuO nanoparticles in the preparation of CuO/CPE, CV was used to investigate the influence on the electrochemical responses of olivetol solution [30]. The CV curves and trends are disclosed in Figure 2a,b. The peak current increased with the proportion of CuO nanoparticles from 5% to 10% and then decreased at a greater proportion (from 15% to 20%). This might be because of the augment in the number of active sites in CPE introduced by the addition of CuO nanoparticles. However, the excess addition of CuO nanoparticles in the paste would lead to a decrease in conductivity [31]. Therefore, 10.0% (*w*/*w*) of CuO nanoparticles were adopted through the preparation of CuO/CPE and p-L-serine/CuO/CPE.

The electropolymerization of L-serine was carried on the surface of CuO/CPE using cyclic voltammetry in the existence of 0.1 mol L^−1^ NaOH and cyclically scanned between −0.6 to 1.8 V for 20 cycles. As elucidated in Figure 2c, the peak current in the CV plot gradually changed with increasing cycles, which endorsed the growth of polymeric films on the electrode. The electropolymerization cycles of obtained p-L-serine/CuO/CPE were investigated by changing the cycle numbers from 4 to 24 cycles (Figure 2d) and submitted to the electrochemical detection of olivetol. It could be observed that the peak current was enhanced up to 20 cycles and decreased slightly after that (the CV curves were provided in Figure S1). The p-L-serine membrane was formed and deposited on the surface of CuO/CPE, and the thickness of the layer might affect the electrocatalytic property of the forming p-L-serine/CuO/CPE, which was closely related to the electropolymerization cycles [24]. Therefore, 20 cycles of electropolymerization were selected as the optimum condition for the fabrication of the p-L-serine/CuO/CPE. After electropolymerization, the modified electrode was carefully rinsed with water and stored for further use.

### 3.3. Electrochemical Characterization of the p-L-Serine/CuO/CPE

**Figure 3:**
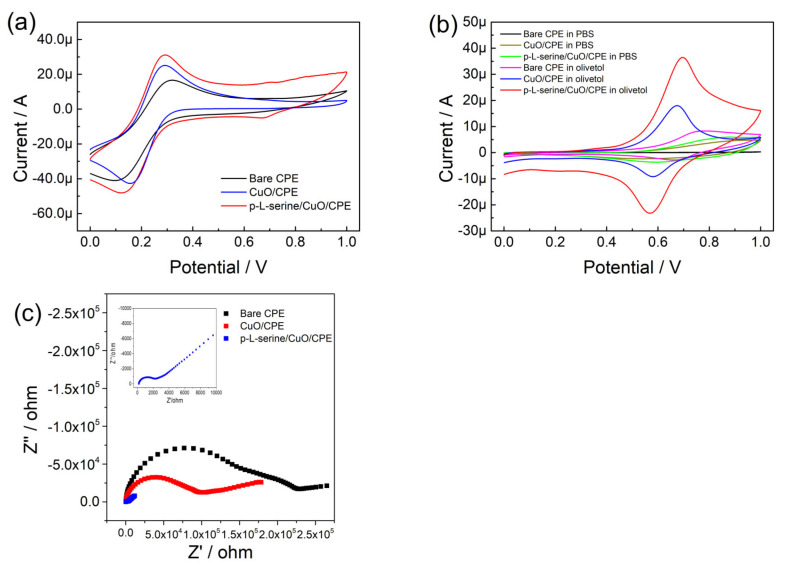
(**a**) CV curves of potassium ferricyanide on bare CPE, CuO/CPE, and p-L-serine/CuO/CPE; (**b**) CV curves of PBS solution and olivetol on bare CPE, CuO/CPE, and p-L-serine/CuO/CPE; (**c**) Nyquist plots of bare CPE, CuO/CPE, and p-L-serine/CuO/CPE.

The electrochemical responses of the bare CPE, CuO/CPE, and p-L-serine/CuO/CPE were studied in 5 mmol L^−1^ of potassium ferricyanide containing 0.1 mol L^−1^ of KCl as the supporting electrolyte. A comparison of the corresponding peak currents in CV is given in Figure 3a. The peak currents of potassium ferricyanide at both CuO/CPE and p-L-serine/CuO/CPE increased as compared to those at the bare CPE. Though the potential difference of p-L-serine/CuO/CPE was bigger than that of CuO/CPE, the response was also higher. The result showing the modification of CPE enhanced the electrochemical activity of the electrode. Then, the responses of modified electrodes on PBS and olivetol solutions were further compared. As shown in Figure 3b, both bare CPE and modified CPE had no response in CV curves (black, brown, and green plots). Meanwhile, a couple of redox peaks could be found in CuO/CPE and p-L-serine/CuO/CPE when analyzed in olivetol solution, which was located at around 0.65 V (blue and red plots). According to these characterizations, p-L-serine/CuO/CPE could be used in the electrochemical analysis of olivetol by enhanced signals.

The EIS plots of bare CPE, CuO/CPE, and p-L-serine/CuO/CPE were investigated to find the change of charge transfer resistance (Rct) of electrodes during modifications. The diameter of the semicircle in the Nyquist plots reflected the value of Rct, and the diameters of modified electrodes were smaller than that of bare CPE, indicating a decrease in resistance [23]. Through the fitting of EIS data with the equivalent circuit, the values of Rct were stimulated as 130 KΩ, 66 KΩ, and 1896 Ω for bare CPE, CuO/CPE, and p-L-serine/CuO/CPE, respectively. The change of Rct values was in accordance with that of the diameters in the Nyquist plots. Moreover, the effective area of p-L-serine/CuO/CPE was calculated by the Randles-Sevcik equation (I_p_ = 2.69 × 10^5^ A D^1/2^ n^3/2^ υ^1/2^ C), where A is the effective area (cm^2^), D is the diffusion coefficient of potassium ferricyanide (7.6 × 10^−6^ cm^2^ s^−1^), n is the transfer number of electrons (1), υ is the scan rate (0.05 V s^−1^), and C is the concentration of potassium ferricyanide (5 mmol L^−1^) [32]. In this study, the effective area of p-L-serine/CuO/CPE was 0.039 cm^2^.

### 3.4. Effect of pH

**Figure 4:**
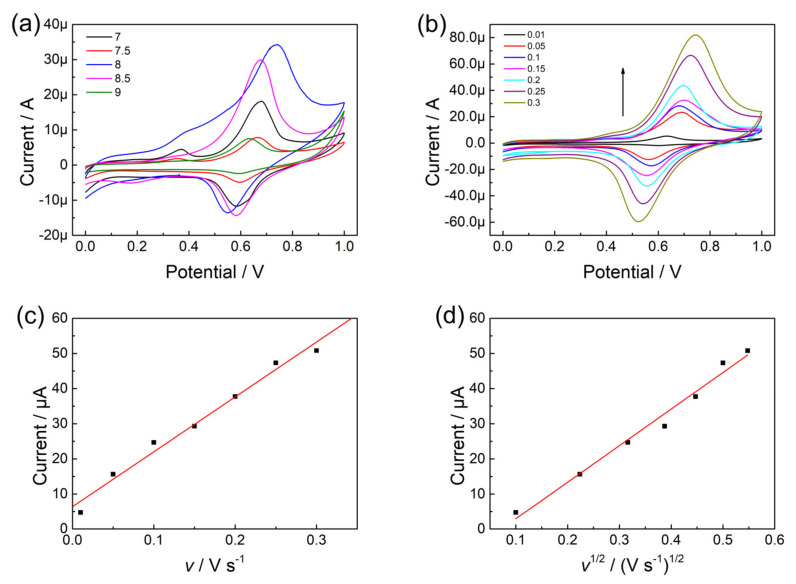
(**a**) Effect of pH on CV curves of olivetol; (**b**) CV curves of p-L-serine/CuO/CPE in olivetol at different scan rates; (**c**) The linear graph of peak currents and scan rates; (**d**) The linear graph of peak currents and (scan rates)^1/2^.

To investigate the experimental conditions in terms of the pH of supporting electrolytes, various pH of supporting electrolytes (7.0, 7.5, 8.0, 8.5, and 9.0) containing olivetol were electrochemically detected in CV using p-L-serine/CuO/CPE (Figure 4a). The results showed that the pH affected the peak currents of olivetol. The peak current of olivetol increased with increasing pH from 7.0 to 8.0, and then higher pH decreased the peak current sharply. Thus, the pH of the supporting electrolyte was adjusted to pH 8.0 for the following experiments.

### 3.5. Effect of Scan Rate

The influence of scan rates on the peak current of olivetol (pH 8.0) was studied using p-L-serine/CuO/CPE in the CV method. Figure 4 demonstrated the resulting CV curves at scan rates varying from 0.01 V s^−1^ to 0.3 V s^−1^. and the corresponding linear plots of peak currents and square roots of scan rates. As shown in Figure 4b, the oxidation peak potentials slightly shifted to positive values with the increasing scan rate, and the peak currents increased as well. With the increase in scan rate, the peak current increased linearly (Figure 4c), and the slope was 156.2 µA s V^−1^ (r^2^ = 0.979). Moreover, the plot in Figure 4d demonstrated the linear relationship between the peak current and the square root of the scan rate, which would be expressed as Ip = 104.1 v^1/2^ − 7.45 (r^2^ = 0.981). These trends suggested that the electrode reaction on the p-L-serine/CuO/CPE is controlled by diffusion as a rate-determining step [33].

**Figure 5:**
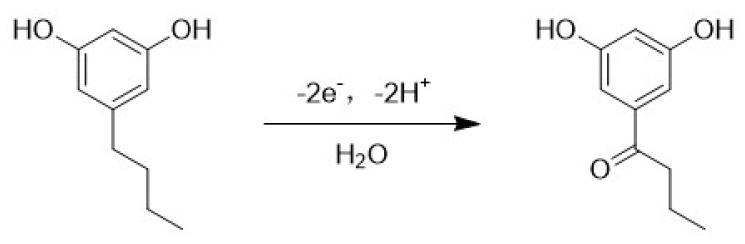
Possible mechanism of the electrooxidation of olivetol.

As reported in S.T. Nandibewoor, the number of electrons involved in the reaction was 2, which was equal to the number of protons. Based on these earlier papers, a possible mechanism is shown in Figure 5.

### 3.6. Analytical Performance of p-L-Serine/CuO/CPE

**Figure 6:**
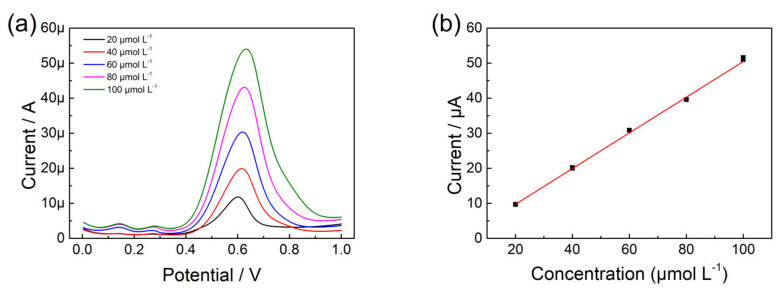
(**a**) The DPV of p-L-serine/CuO/CPE in different concentrations of olivetol (pH 8.0); (**b**) Plot of peak current versus concentration of olivetol.

As a sensitive electrochemical method, the DPV measurements were studied for the determination of olivetol under optimum conditions. Figure 6 provided the DPV plots and calibration curves under increasing concentrations ranging from 20 to 100 μmol L^−1^. It could be found that the oxidation peak appeared at 0.6 V as well, and the peak current increased with increasing concentrations of olivetol. After fitting and calculation, the DPV response of olivetol expressed good linearity, which could be explained as Ip = 0.514C − 0.519 with r^2^ = 0.999. The Limit of detection (LOD) and quantification (LOQ) was calculated as 3 times S/N and 10 times S/N, where N is the slope of the standard curve and S is the standard deviation of blank currents [34]. The LOD and LOQ in this study were calculated as 1.04 and 3.47 μmol L^−1^. The existing reported electrochemical detection of olivetol was reported by S.T. Nandibewoor in 2017 [35]. Some of the parameters in the method were compared in Table 1. Although the reported gold electrode had good sensitivity for olivetol (0.1–1.5 μmol L^−1^), the proposed p-L-serine/CuO/CPE demonstrated good linearity and a wide quantitative analysis range from 20 to 100 μmol L^−1^. The sensitivity was a typical advantage of the gold electrode. And many gold sensors and modified gold electrodes were applied to the trace level of target samples. However, the advantages of CPE were cheap and easy, which could enlarge the application ranges. Considering the materials and preparation of the modified CPE, this sensor could be applied in the rapid analysis in the field and cost control.

### 3.7. Reproducibility, Repeatability, Stability, and Interference Study

**Figure 7:**
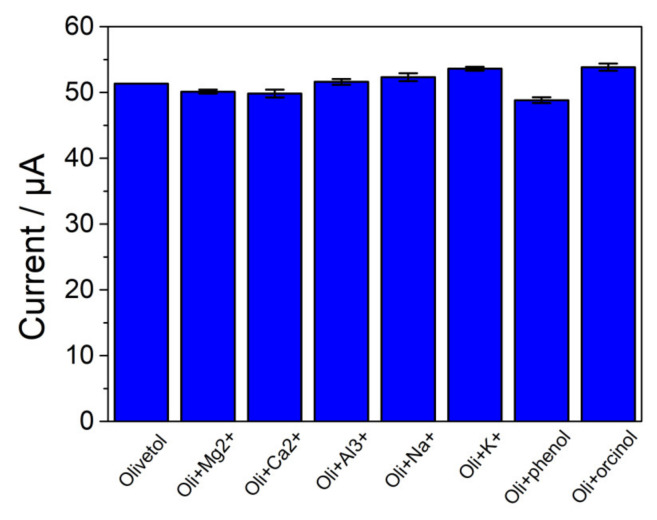
Peak current of p-L-serine/CuO/CPE in olivetol containing various interfering substances.

For the reproducibility test, five independently modified electrodes were used to determine the same sample and the resulting relative standard deviation (RSD) was 2.57%. The repeatability of the modified electrode was obtained by testing five times a day, and the obtained RSD was 2.42%. For the stability test, the modified electrode was stored in the laboratory at room temperature, and the signal only decreased by 3.00% of its initial value over a week. The interference study of the modified electrode was studied in the presence of interferents. The concentrations of inorganic ions and organic compounds were 10-fold higher than that of the target compound, and the concentration of orcinol was the same as that of the target compound. It was found that the addition of Mg^2+^, Ca^2+^, Al^3+^, Na^+^, K^+^, phenol and orcinol influenced the signal in bearable degrees in the determination of olivetol (Figure 7). The relative errors during the test were kept at less than 5.0%, indicating that these compounds were not potential interferences for this analysis [36].

## 4. Conclusions

In this study, CuO nanoparticles and L-serine were modified on CPE, resulting in the p-L-serine/CuO/CPE for the sensitive detection of olivetol in the electrochemical method. The morphology of CuO nanoparticles was well characterized, and the modification of p-L-serine by electropolymerization was observed by comparing it to bare CPE. Through the optimization of fabrication and detection parameters, the fabrication of p-L-serine/CuO/CPE was performed in 10.0% (*w*/*w*) of CuO nanoparticles and 20 cycles of polymerization, and the detection was utilized in buffer solution at pH 8.0. Under the optimized conditions, the proposed sensor showed a good linear quantitative analysis ability and a wide analysis range. The reproducibility, repeatability, stability, and anti-interference ability ensured the practical ability and application prospect of this sensor. However, the sensitivity and specificity of the sensor still need to investigate based on the modification of CPE, which deserves more research and investigation.

## Figures and Tables

**Table 1:** Comparison of reported sensors for the detection of olivetol.

Electrode	Method	Linear Rage (μmol L^−1^)	LOD (μmol L^−1^)
Gold electrode	SWV	0.1–1.3	0.0048
	DPV	0.1–1.5	0.0019
p-L-serine/CuO/CPE	DPV	20–100	1.04

SWV: Square wave voltammetry.

## Data Availability

The data presented in this study are available on request from the corresponding authors. The data are not publicly available due to security requirements in the institute of the corresponding author.

## Supplementary Materials

The following supporting information can be downloaded at: https://www.mdpi.com/article/10.3390/nano13010070/s1, Figure S1: CV of p-L-serine/CuO/CPE prepared by different electropolymerization cycles.
