# Supplementary figures and images for: Electrochemical Detection of Olivetol Based on Poly(L-Serine) Film Layered Copper Oxide Modified Carbon Paste Electrode (p-L-Serine/CuO/CPE)
Source: Nanomaterials (Basel). 2022 Dec 23;13(1):70. doi: 10.3390/nano13010070 (PMC9824513; doi:10.3390/nano13010070)

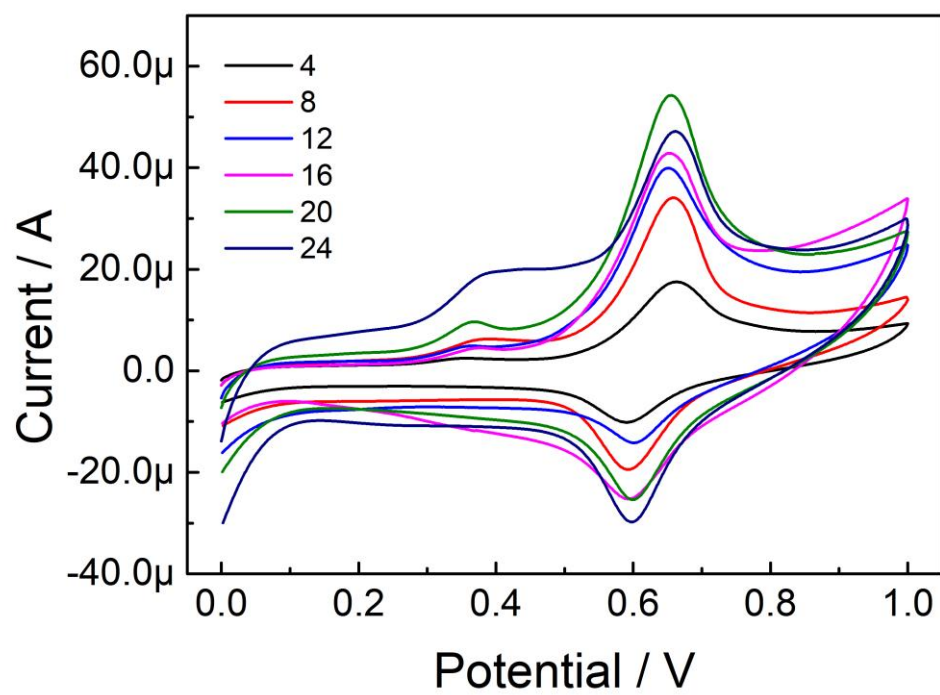

**Figure S1.** CV of p-L-serine/CuO/CPE prepared by different electropolymerization cycles.

Supplement: Supplementary file 1 [file nanomaterials-13-00070-s001.zip › nanomaterials-2080866-supplementary.pdf]
